# A new paradigm for cancer immunotherapy: targeting immunogenic cell death-related noncoding RNA

**DOI:** 10.3389/fimmu.2024.1498781

**Published:** 2025-01-23

**Authors:** Guojuan Sun, Ling He

**Affiliations:** The Ward Section of Home Overseas Doctors, Hospital of Chengdu University of Traditional Chinese Medicine, Chengdu, China

**Keywords:** ncRNAs, DAMPs, immunogenic cell death, antitumor immune responses, cancer immunotherapy

## Abstract

Cancer immunotherapy has shown significant potential in treating several malignancies by stimulating the host immune system to recognize and attack cancer cells. Immunogenic cell death (ICD) can amplify the antitumor immune responses and reverse the immunosuppressive tumor microenvironment, thus increasing the sensitivity of cancer immunotherapy. In recent years, noncoding RNAs (ncRNAs) have emerged as key regulatory factors in ICD and oncologic immunity. Accordingly, ICD-related ncRNAs hold promise as novel therapeutic targets for optimizing the efficacy of cancer immunotherapy. However, the immunomodulatory properties of ICD-related ncRNAs have not yet been comprehensively summarized. Hence, we summarize the current knowledge on ncRNAs involved in ICD and their potential roles in cancer immunotherapy in this review. It deepens our understanding of ncRNAs associated with ICD and provides a new strategy to enhance cancer immunotherapy by specifically targeting the ICD-related ncRNAs.

## 1 Introduction

Over the past few decades, there has been a prospective development in cancer treatment, mainly oriented towards immunotherapy, replacing or combining with classical treatment regimens (1). Cancer immunotherapies mainly include immune checkpoint inhibitors, therapeutic vaccines, and adoptive cell therapies, all of which have been successfully implemented in clinical settings (2–6). However, only a small subset of patients benefit from cancer immunotherapies based on these strategies, while other patients experience low response rates or suffer from immunotherapy-related toxicities (7, 8). Therefore, advanced strategies to break the existing bottleneck of cancer immunotherapy are urgently required.

Immunogenic cell death (ICD) is a modality of regulated cell death triggered by cytotoxic treatments, which serves as the initial signal for the antitumor immune responses in immunocompetent hosts (9). It involves the release of damage-associated molecular patterns (DAMPs) and tumor-associated antigens (TAAs) from stressed or dying cells, which enhances antitumor immune responses (10). In cancer immunotherapy, ICD not only eliminate cancer cells directly but also transforms dying tumor cells into a source of antigens, thereby activating dendritic cells and cytotoxic T-cell responses (11, 12). Furthermore, ICD promotes long-lasting antitumor immune responses by reversing the immunosuppressive tumor microenvironment and synergizing with immune checkpoint inhibitors, making it a cornerstone for developing next-generation cancer immunotherapies (9). Accordingly, the concept of ICD reveals the immunogenic capacity of cancer cells and introduces a new paradigm in cancer immunotherapy (13).

**Figure 1:**
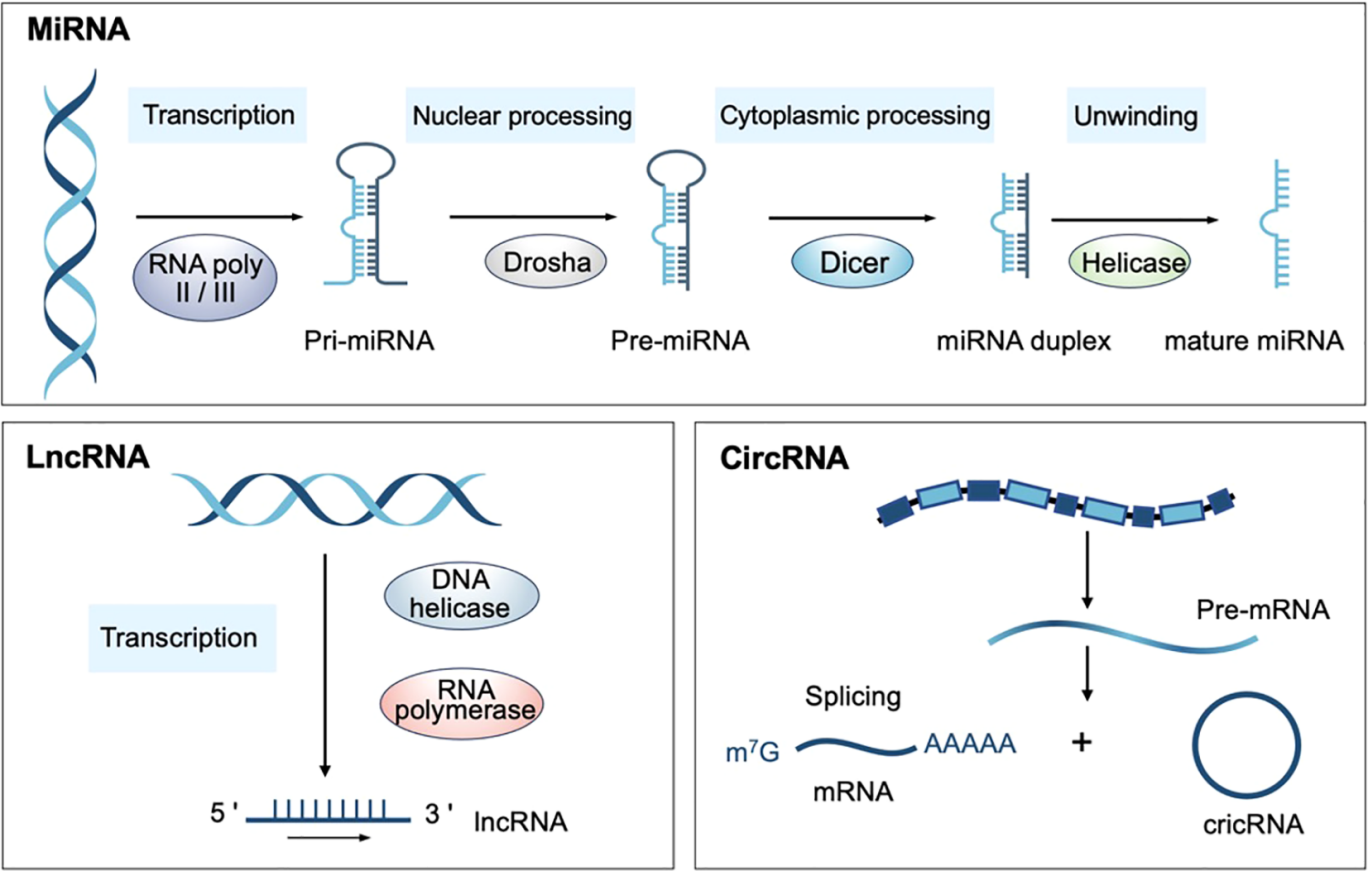
Biosynthesis of ncRNAs. (miRNA, MicroRNA; lncRNA, Long non-coding RNA; circRNA, Circular RNA).

To date, shreds of evidence have manifested that noncoding RNAs (ncRNAs) are the hubs of ICD-related molecular communication networks (14, 15). NcRNAs refer to those RNA molecules that do not participate in protein-coding and have been revealed to regulate various cellular processes in both developmental and pathological conditions (16). NcRNAs are categorized into long noncoding RNAs (lncRNAs), microRNAs (miRNAs), circular RNAs (circRNAs), small interfering RNAs (siRNAs), and Piwi-interacting RNAs (piRNAs) based on their length, size, shape, and cellular functions (17). Among these, miRNA, lncRNA and circRNA are the main types of ncRNA that exert multiple effects on the complex regulation of ICD and antitumor immune response (**Figure 1**) (16). Mature miRNAs are single-stranded ncRNA with a length of approximately 22 nucleotides and participate in antigen presentation, immune cell infiltration, and endoplasmic reticulum stress-mediated ICD amplification (18). LncRNAs, a subset of regulatory ncRNAs with a length of more than 200 nt, serve as hinges bridging ICD and antitumor immunity, and can serve as prognostic biomarkers in hepatocellular carcinoma and lung adenocarcinoma (19–21). CircRNAs are endogenous ncRNAs characterized by a covalently closed loop structure, lacking the 3′poly(A) tail and the 5′-cap, which modulate metabolic reprogramming and reshape the immune landscape (22). In cancer immunotherapy, ICD-related ncRNAs regulate important pathways involved in ICD, including the release of DAMPs, the recruitment of immune cells, and the reprogram of the tumor immune microenvironment (18, 23). Therefore, targeting ICD-related ncRNAs can enhance the immunogenicity of tumor cells, overcome resistance to conventional therapies, and improve the effectiveness of classical immunotherapies such as anti-PD-1 immunotherapy. This innovative strategy offers a promising opportunity for developing personalized and effective cancer immunotherapies.

Taken together, emerging evidence reveals the untapped potential of ncRNAs in inducing ICD and triggering antitumor immune responses. Nevertheless, there is a lack of systematic elaboration on the immunomodulatory properties of ICD-related ncRNAs. Consequently, this review aims to systematically summarize ICD-related ncRNAs, elaborate the regulatory network underlying ncRNA involvement in ICD, and explore their prospective functions in cancer immunotherapy. We hope this review will deepen our understanding of ICD-related ncRNAs and provide new strategies to maximize the efficiency of cancer immunotherapy by specifically targeting the ICD-related ncRNAs.

## 2 Outward extension of ICD in cancer immunotherapy

**Figure 2:**
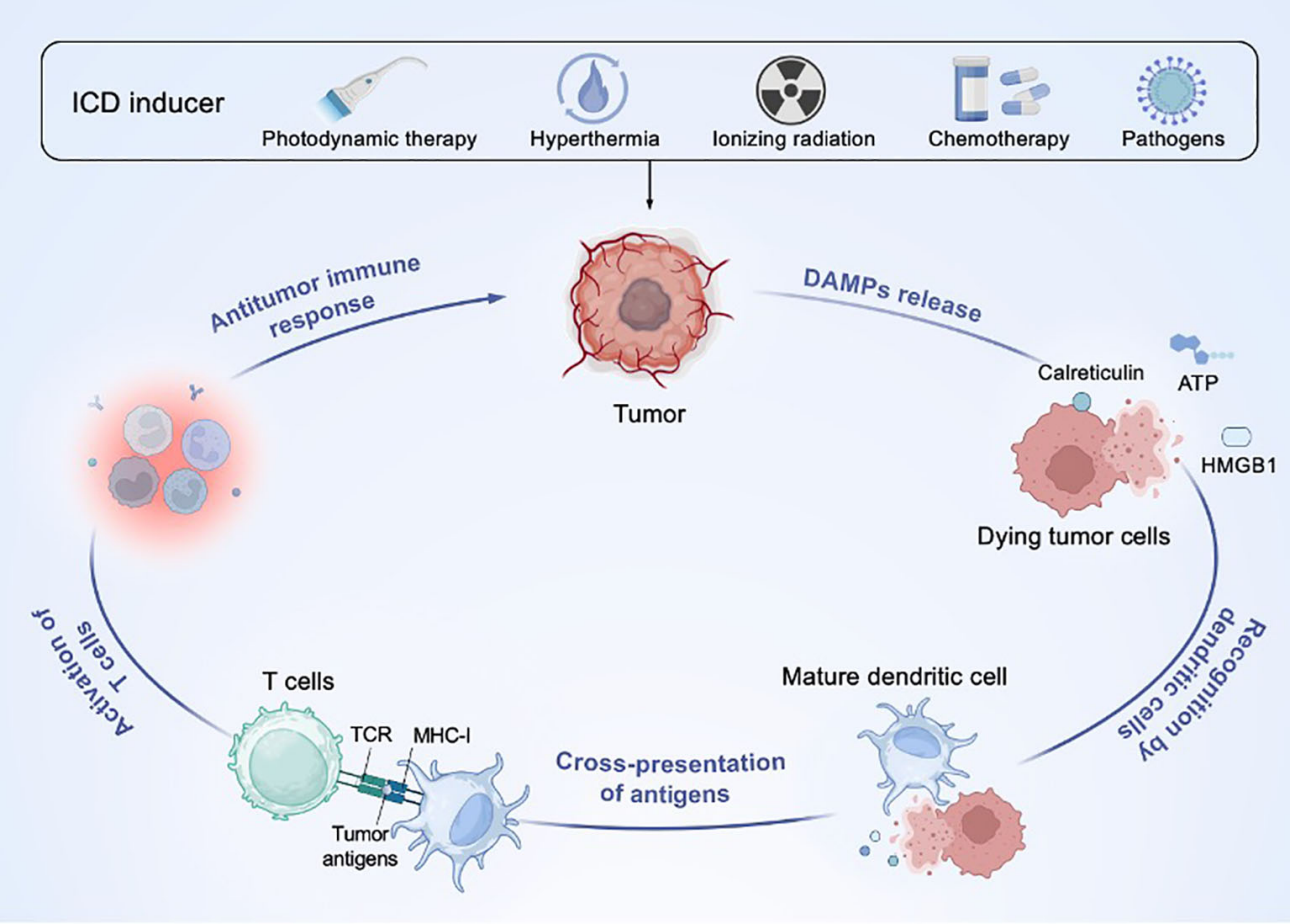
Mechanism underlying ICD in cancer immunotherapy. ICD inducers induce dying tumor cells release damage-associated molecular patterns (DAMPs), such as adenosine triphosphate (ATP), and high-mobility group box 1 (HMGB1), which activate dendritic cells (DCs), T cells and other immune cells, promoting antigen presentation and antitumor immune response. TCR, T cell receptor; MHC, major histocompatibility complex class I.

Stressed or dying cells release cytokines and damage-associated molecules that interact with organisms to indicate the immunogenicity of cell death (24). In the absence of reactive epitopes, these signaling molecules may drive apoptosis rather than adaptive immunity; however, in the case of malignant cells with sufficient antigenicity, they execute an immunogenic death mode, leading to cytotoxic lymphocyte-mediated immune response. This suggests that ICD inducers provide new opportunities for cancer immunotherapy. In cancer immunotherapy, ICD inducers stimulate tumor cells to release signals that activate resident immune cells and recruit inflammatory cells, thereby enhancing the antitumor immune response (**Figure 2**) (25). Simultaneously, numerous DAMP signals (such as calreticulin and high mobility group protein 1), inflammatory factors (such as interferons and interleukins) and tumor antigens are present in a spatiotemporally dependent manner, corresponding to the specific antitumor immune response (26).

The ability to initiate ICD is contingent upon the capacity to induce endoplasmic reticulum (ER) stress, as the exposure of ER chaperones to the outer plasma membrane is a fundamental event in ICD induction (24). Type I ICD inducers (e.g., chemotherapy) initiate ICD through non-ER pathways but prompt ICD-related immunoreactivity through secondary or “side” effects of ER stress; type II ICD inducers (e.g., photodynamic therapy) selectively target ER components to induce ICD by directly mediating ER stress (27). Under the process of ICD inducers, damage-associated molecular patterns (DAMPs) promote conversion in the tumor microenvironment from a non-inflammatory “cold” immune state to an inflammatory “hot” immune state by coordinating elaborate information transmission between cancer cells and immune cells (26).

According to prior research, DAMPs exposure trigger ICD by functioning as “find-me,” “eat-me,” and “danger” signals (24). (1) “find-me” signal: it mainly refers to adenosine triphosphate (ATP) released by tumor cells, which recruits macrophages, etc., to seek out damage sites, leading to the secretion of pro-inflammatory cytokines and activation of subsequent adaptive immunity; (2) “eat-me” signal: it mainly refers to the exposure of the ER molecular chaperone calreticulin on the surface of dying cells, which increases the phagocytosis and cross-presentation of tumor antigens in the participation of major histocompatibility complex class I (MHC-I) to activate antigen-specific T cell responses; (3) “danger” signals: it mainly refers to high mobility group protein 1 (HMGB1) released by tumor cells, which increases the binding affinity of transcription factors and DNA by bending or twisting the DNA double helix, regulates the immunogenicity of tumor cells, and promotes the subsequent inflammatory response and ICD. In addition, recent studies suggest that mitochondrial DNA (mtDNA) functions as a DAMP signal, connecting cellular stress to ICD-mediated antitumor immunity (28, 29). Under conditions of cellular stress, mtDNA is released into the cytoplasm and induces ICD through several pathways, including the cGAS-STING signaling pathway, toll-like receptor 9 (TLR9), and the NOD-like receptor family pyrin domain-containing 3 (NLRP3) inflammasome, thereby enhancing the immune response (30; Wang et al., 2023). In short, the DAMPs series signals released by tumor cells are crucial for initiating and expanding the ICD cascade.

The first event of the ICD cascade is the exposure of calmodulin to the surface of dying cells, which is commonly located in the lumen of ER and translocates within hours of ICD inducer stimulation (31). Subsequently, another hallmark of ICD that can be observed after calmodulin exposure is the translocation of heat shock proteins (HSPs), which triggers the activation and maturation of DCs (32). After 12 hours of exposure to calmodulin, the second event of the ICD cascade started. HMGB1 is released into the extracellular space and binds to Toll-like receptors (TLRs), ensuring the optimal processing and presentation of tumor antigens by DCs (33). The final event in the process of ICD is the secretion of ATP into the extracellular space, which serves as a “find-me” signal (24). Dying cells mark their presence with “find-me” signals so that phagocytes can quickly recognize and efficiently eliminate them. Consequently, this immunological cascade specifically activates cytotoxic T cells to induce immunogenic death against cancer cells and reprograms the “immune infiltration” microenvironment, which improves the clinical effect of cancer immunotherapy (11).

Emerging evidence suggests that ncRNAs with immunogenic properties are involved in multiple processes of T cell-dependent ICD (34). ICD-related ncRNAs can recruit cytotoxic T cells to the tumor site and participate in the reprogramming of the “immune infiltration” microenvironment (35). Therefore, ICD-related ncRNAs possess the capability to optimize the clinical efficacy of cancer immunotherapy by reversing the immunosuppressive system and amplifying immunogenic effects.

## 3 Crosstalk and communication between ncRNAs and ICD

### 3.1 DAMPs as bridges for communication

**Figure 3:**
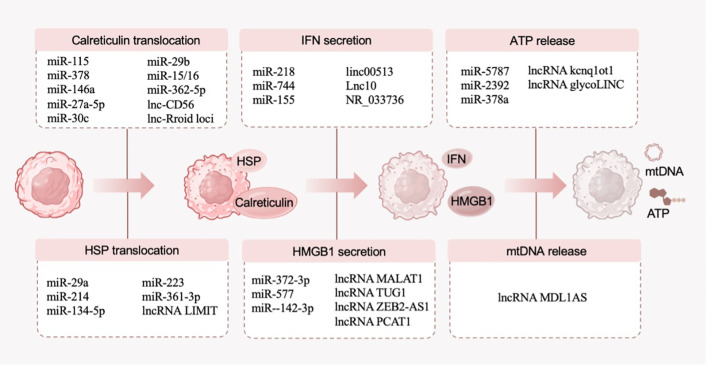
DAMPs (calreticulin, HSP, HMGB1, ATP, type I IFNs and mtDNA) in the ICD process and their related ncRNAs.

DAMPs are messenger molecules emitted by stressed dying cells, fulfilling the role of “danger signal” or immune adjuvant (36). While undergoing ICD, the released DAMPs efficiently enhance the immunogenicity of cells and are recognized by innate pattern recognition receptors (PRRs), thus facilitating the recruitment and maturation of antigen-presenting cells and activating adaptive immune responses against neoplastic antigens (11). With the broadening of the research on DAMPs, the molecular types of DAMPs are constantly expanded. At present, the molecules that have been encompassed in the category of DAMPs include calreticulin, heat-shock protein (HSP), HMGB1, ATP, type I interferons (IFNs) and mitochondrial DNA (mtDNA), all of which are closely related to ICD (36). In this section, we provide an overview of only a few DAMPs that have been extensively studied and discuss the related ncRNAs (**Figure 3**).

#### 3.1.1 Calreticulin and ncRNAs

Calreticulin is an evolutionarily conserved DAMP that resides in the lumen of the ER (37). As an essential ER molecular chaperone, it is closely related to Ca^2+^ homeostasis, assembly of MHC-I molecules, antigen loading and immunogenic cell death (38). When cells are exposed to stress factors such as ICD inducers, calreticulin is transferred to the cell membrane surface, where it acts as an “eat me” signal to interact with CD91 and TLR4 for promoting phagocytosis mediated by antigen-presenting cells (APCs), ultimately triggering the cross-presentation of antigens and the initiation of T cell-dependent antitumor immune response (13). It has been confirmed that calreticulin exposure is a prerequisite for ICD and the subsequent specific oncologic immunity, suggesting that calmodulin-related ncRNAs are potential targets for cancer immunotherapy.

Truxova et al. found that calreticulin exposure increases the aggregation of CD11c^+^CD14^+^ marrow cell populations in tumor microenvironment, thereby enhancing the phagocytic and cytotoxic effects of natural killer (NK) cells on acute myeloid leukemia cells (39). From the viewpoint of epigenetic mechanisms, many ncRNAs (including miR-115, miR-378, miR-146a, miR-27a-5p, miR-30c, miR-29b, miR-15/16, miR-362-5p, lnc-CD56 and lnc-Rroid loci) are involved in the process of NK cells-mediated immune response (40). According to these observations, a probable signaling overlap between ncRNAs and calreticulin- mediated ICD is implied.

#### 3.1.2 HSP and ncRNAs

HSPs (mainly HSP70 and HSP90) are also typical DAMPs and are considered to be important signals that mediates the ICD of cancer cells (41). HSPs are exposed on the cell surface in the form of a high-immunogenicity complex at the early stage of ICD and dictates the immunogenicity of cancer cells (42). Donatella et al. found that the high expression of HSP under stress promotes the cross-presentation function of APCs, thus enhancing immunogenicity (43). Furthermore, HSP plays a chemokine-like role in recruiting immune cells (44). It promotes DCs to secrete pro-inflammatory cytokines such as IL-6, IL-12, and granulocyte-macrophage colony-stimulating factors, further strengthening the ICD-induced immune response (44).

Multiple lines of evidence indicate that ncRNAs are relevant to HSP, revealing the importance of ncRNAs in immunogenicity and anti-tumor immune response (45). Regarding HSP-related miRNAs: miR-29a and miR-214 are positively correlated with HSP90; miR-134-5p, miR-223, and miR-361-3p are negatively correlated with HSP90. These results indicate that HSP-related ncRNAs fulfill a multifaceted role in ICD, suggesting that targeted strategies should be designed according to actual clinical conditions. On the subject of lncRNAs, LIMIT (lncRNA inducing MHC-I and immunogenicity of tumor) disrupts the effects of HSP and leads to the subsequent transcription of the MHC-I machinery, so it is claimed to have a cancer immunogenic function (46). Accordingly, LIMIT and other HSP-related ncRNAs conceptually show therapeutic promise in ICD-mediated cancer immunotherapy.

#### 3.1.3 HMGB1 and ncRNAs

HMGB1 is a widely expressed non-histone nuclear protein that participates in maintaining nucleosome structure and regulating genetic processes (47). What’s more, studies have shown that the release of HMGB1 from the nucleus into the extracellular fluid of pre-apoptotic or dying cells is one of the prominent attributes of ICD (48). During the ICD process, the translocated HMGB1 couples with TLR4 on the surface of DCs to improve the efficient processing and cross-presentation of tumor-associating antigens before apoptosis, thereby activating specific T-cell immune responses (49). In cancer immunotherapy models, knocking out HMGB1 in tumor cells or neutralizing HMGB1 with specific antibodies can notably reduce the tumor-suppressing capacity (50). From a clinical perspective, interfering with the functions of HMGB1 renders breast cancer patients with TLR4 allele loss more susceptible to relapse after treatment (51).

Considering the significant role of HMGB1 in the process of ICD, HMGB1-related ncRNAs are regarded as potential targets for inducing oncologic immunogenicity. For example, lncRNA MALAT1, lncRNA TUG1, lncRNA ZEB2-AS1, lncRNA PCAT1, miR-372-3p, miR-577 and miR–142-3p are upstream targets of HMGB1, suggesting that these ncRNAs are associated with ICD (52). Among these, miR-142-3p, which regulates HMGB1 formation, has been shown to fulfill an important immunomodulatory role in various cancers, such as breast cancer, lung cancer, colorectal cancer and gastric cancer (53). LncRNA OIP5-AS1 was demonstrated to reduce inflammation by upregulating HMGB1, and lncRNA ZEB2-AS1 depletion was demonstrated to inhibit the invasion of pancreatic cancer cells by suppressing HMGB1 (54, 55). These results point to new possibilities: (1) ncRNAs associated with HMGB1 and ICD can serve as therapeutic targets for tumors; (2) cascade amplification of ICD effects can be achieved by targeting ncRNAs, thereby expanding tumor immunotherapy.

#### 3.1.4 ATP and ncRNAs

When ICD occurs, ATP is secreted from dying cells in an autophagy-dependent and pannexin-1-mediated manner (56). The secreted ATP is an important indicator that mediates ICD and serves as an “eat-me” signal to swiftly recruit DCs and macrophages to the neoplastic region (57). In detail, ATP binds to purinergic receptor P2Y2 (P2RY2) on the cellular surface, promoting the infiltration of tumor-infiltrating myeloid cells into the neoplastic area (57). In addition, the released ATP by dying tumor cells fosters the formation of caspase-1-dependent NLRP3 inflammasome in DCs and promotes the secretion of IL-1β, further stimulating antitumor immune responses (13). It is worth noting that although extracellular ATP plays an essential role in the ICD process, non-immunogenic cell death patterns also lead to the release of ATP. Hence, the release of ATP cannot be used as the exclusive indicator of ICD, and it needs to be combined with other DAMPs to monitor the occurrence of ICD more accurately.

ATP is an essential substance in cells, and the molecular regulatory network related to ATP is extremely complex (58). Consequently, ncRNAs are extensively implicated in the multifaceted network of ATP. For example, mitochondrial ncRNAs such as miR-5787, miR-2392, lncRNA MDL1, LIPCAR are directly involved in the production of ATP in mitochondria; lncRNA kcnq1ot1 and miR-378a regulate ATP synthase through the competing endogenous RNAs (ceRNA) axis in type 2 diabetic heart; and lncRNA glycoLINC enhances glycolytic flux to increase ATP production (59–61). It is speculated that ATP-related ncRNAs may exert immunostimulatory effects in cancer immunotherapy through the DAMP-ICD axis. Disconsolately, there is no convincing evidence to straightforwardly indicate that ncRNAs induce ICD by ATP-mediated immunogenicity, and further research is needed to explain this issue.

#### 3.1.5 Type I IFNs and ncRNAs

Tumor cells undergoing ICD secrete type I IFNs through the activation of TLR3 by cyclic guanosine monophosphate-adenosine monophosphate synthase/stimulator of interferon genes (cGAS/STING) signaling pathway (62). The secreted type I IFNs can couple with homodimeric or heterodimeric receptors expressed on the surface of immune cells and exert immunostimulatory effects (63). In addition to direct immunostimulatory effects, type I IFNs can couple with IFN-α and IFN-β receptors on the surface of tumor cells, triggering the recruitment of T cells in the tumor by autocrine and paracrine signaling pathways (64). Moreover, studies have found that the induction of ICD in tumors driven by anthracyclines and radiotherapy are forcefully dependent on type I IFNs (65). Therefore, type I IFNs and their relevant indicators can be utilized to evaluate the ICD of tumor cells and predict the clinical response of anthracycline-based chemotherapy.

Type I IFNs have been evidenced to have functional interactions with miR-218, miR-744, miR-155, linc00513, Lnc10, and NR_033736, suggesting that these ncRNAs are involved in the induction of ICD in tumors (66). Given that type I IFNs are considered notable ICD inducers for anthracycline-based chemotherapy, type I IFNs-related ncRNAs have received widespread attention as sensitizing or prognostic molecules for anthracycline-based chemotherapy.

#### 3.1.6 mtDNA and ncRNAs

Mitochondria are specialized cellular organelles that contain one or more copies of their own genome (mtDNA) (67). When mitochondria are damaged or stressed, mtDNA can be released from mitochondria into the cytoplasm and participates in ICD activation as DAMP (28). The main mechanisms by which mtDNA induces ICD in tumor immunotherapy involve the cGAS-STING signaling pathway, toll-like receptor 9, and NLRP3 inflammasome (68). Specifically, (1) mtDNA is recognized by cGAS in the cytoplasm to activate the STING pathway, which then mediates the production of type I IFNs; (2) mtDNA with CpG-rich islands can be recognized by TLR9 to trigger the NF-κB signaling pathway, leading to the release of proinflammatory cytokines such as TNFα and IL-6; (3) mtDNA directly activates the NLRP3 inflammasome to induce the activation of caspase-1, which in turn to promote the release of proinflammatory cytokines (28, 69). Taken together, mtDNA functions as a critical immunoregulatory factor by activating ICD through multiple pathways in response to cellular stress and damage.

In particular, ncRNAs involved in the epigenetic regulation of mtDNA can be encoded by the mitochondrial genome (70). For example, MDL1, which mostly covers the entire mitochondrial D-loop region of the human mtDNA, and MDL1AS, which is the antisense transcript of MDL1, play noteworthy roles in mito-nuclear crosstalk and mtDNA regulation (71). Based on the above background, we support that mtDNA is an important feature of the ICD-corresponded immunogenicity and speculate that mtDNA-related ncRNAs are potential triggers of ICD.

### 3.2 Immunogenic ncRNAs in cancer immunotherapy

The tumor microenvironment can provide a supportive environment for the occurrence of ICD and is a determinant of tumor eradication or survival (72). As a complex and heterogeneous milieu, the tumor microenvironment is composed of surrounding blood vessels, the extracellular matrix (ECM), cancer-associated cells, immune cells, and multiple signaling molecules (73). Among them, the dynamic interaction between ncRNAs and immune cells provides the prerequisite signal for ICD of tumor cells. Hence, this section intends to provide an overview of ncRNAs in the context of the tumor microenvironment and respectively summarize the contributions of ncRNAs in the crosstalk between immune cells and tumor cells, illustrating the relationship of ncRNAs with ICD.

#### 3.2.1 MiRNAs and ICD

Originally, miRNA molecules are transcribed by RNA polymerase II to primary miRNA (pri-miRNA) with a typical stem-loop structure, and then this pri-miRNA is cleaved to a precursor miRNA (pre-miRNA) of approximately 70 bases by Drosha in the nucleus (74). Then, the pre-miRNA is transported into the cytoplasm by exportin5 (Exp5)-ras-related nuclear protein (RAN)-guanosine-5’-triphosphate (GTP) complex and processed to double-stranded miRNA by Dicer (74). One strand of the double-stranded miRNA is degraded, and the remaining “mature” strand assembles with Argonaute to form the RNA-induced silencing complex (RISC) (75). Consequently, the miRNA-RISC is pulled towards complementary sites in the 5’ or 3’ untranslated regions of target mRNAs, resulting in mRNA degradation or translational repression (76). In addition to fulfilling the role of mRNA decoys, miRNAs can also serve as agonists for TLRs, thereby activating downstream effects such as ICD (77).

**Figure 4:**
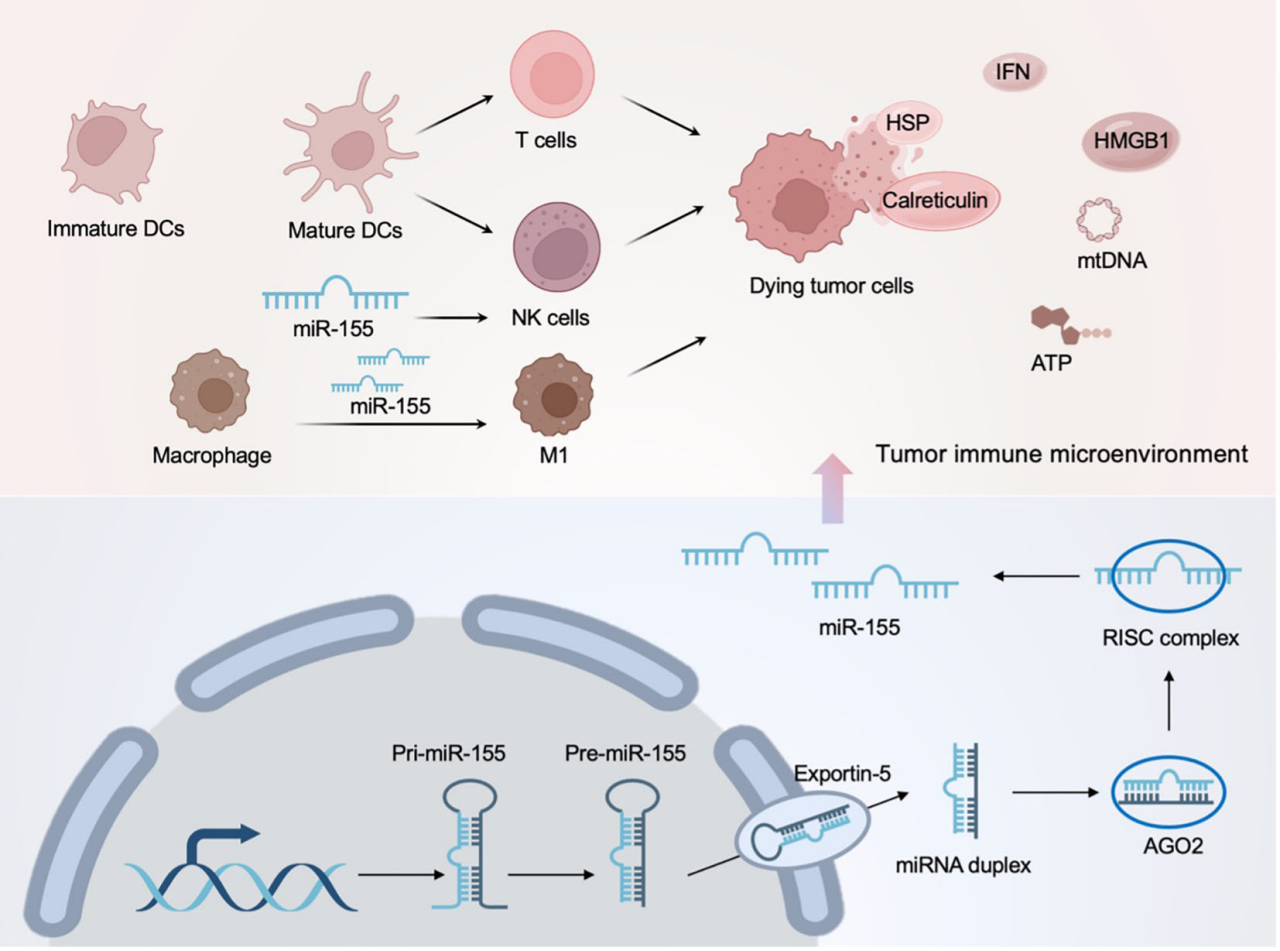
MiR-155 in the tumor immune microenvironment and its mediated ICD regulation.

In the immune microenvironment, NK cells are a type of cytotoxic lymphocyte in the innate immune system that promptly takes action upon identifying “danger” signals (78). NK cells not only have direct cytotoxicity against tumor cells, but also secrete cytokines and chemokines to evoke anti-tumor immune response (79). Furthermore, Kübler et al. have demonstrated that NK cells eradicate ER-stressed cells by calreticulin and receptor NKp46, suggesting that NK cells have a synergistic effect on ICD (80). Studies based on miRNA have shown that miR-30e regulates the cytotoxicity of type I IFN-activated NK cells by targeting perforin (81); highly expressed miR-155 enhances the activity of NK cells and induces the secretion of the immunostimulatory cytokine interferon-γ by regulating hematopoietic-specific inositol phosphatase 1 (82). It is noteworthy that miR-155 is the most-studied miRNA in the immune microenvironment and has been shown to be aberrantly expressed in a variety of tumors, as shown in **Figure 4** (83). In addition to the NK cells mentioned above, miRNA can also regulate macrophages to regain their tumor-killing ability (84). That said, these existing studies of miRNAs in TLRs, immune cells, and the tumor microenvironment at least suggest their therapeutic promise in ICD-mediated cancer immunotherapy.

#### 3.2.2 LncRNAs and ICD

The best-described function of lncRNAs in the tumorigenesis and development of tumors is to regulate gene expression through multiple mechanisms, such as reshaping chromatin and genome architecture, epigenetic regulation, and posttranscriptional processing (85). Moreover, recent studies have shown that lncRNAs not only regulate the recruitment and function of immune cells, such as macrophage polarization and T cell activation, but also regulate cytokines and related pathways, further remodeling the tumor immune microenvironment and activating anti-tumor immune responses (35). For example, lincRNA-Cox2 regulates the expression of many immune-related genes in response to activation of toll-like receptor signaling (86); lnc-DC in the tumor microenvironment regulates the differentiation of DCs and stimulates T cell activation during antitumor immune response (87); overexpression of lncRNA GAS5 amplifies the killing effect of NK cells on liver cancer by regulating miR-544/RUNX3 (88). Based on the above, one possible area for cancer immunotherapy is targeting immunogenic lncRNAs in the tumor microenvironment to initiate ICD.

**Figure 5:**
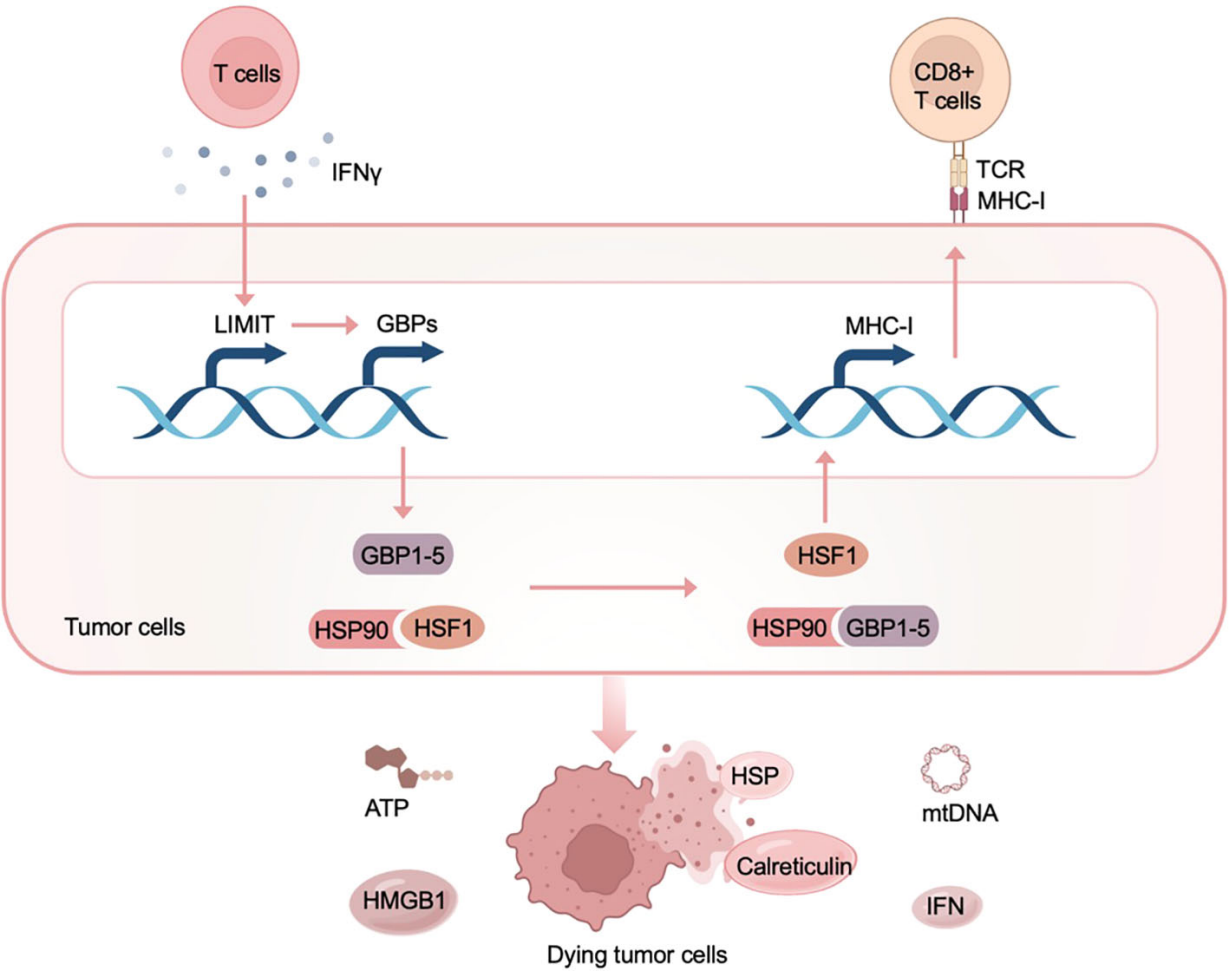
LIMIT mediates ICD as an immunogenic lncRNA in the tumor immune microenvironment. Stimulated by IFNγ, LIMIT cis-activates the guanylate-binding protein (GBP) gene cluster, leading to the subsequent interaction of GBPs with the HSP90, which releases heat shock factor-1 (HSF1) in the cytoplasm and initiates the downstream ICD effect.

Li et al. identify the first immunogenic lncRNA in cancer, establishing a direct link between lncRNAs and ICD for the first time (46). In this study, Li et al. divided melanoma lesions into “hot tumors” with a considerable number of T cell infiltrations and “cold tumors” with only a minor amount of T cell infiltrations, and studied the expression differences of lncRNAs in the two groups. Through functional analysis, one of the lncRNAs enriched in hot tumors was found to have the function of regulating MHC-I expression and tumor immunogenicity, and it was named LIMIT (lncRNA inducing MHC-I and immunogenicity of tumor). Furthermore, clinical analysis showed that the expression level of LIMIT was positively correlated with T cell infiltration, MHC-I antigen presentation, and IFNγ signaling pathway in cancers; patients with high levels of LIMIT expression had improved survival rates and response rates to immunotherapy. In terms of molecular mechanism, LIMIT cis-activates the guanylate binding protein (GBP) gene cluster to bind to HSP90, a type of DAMP, thereby initiating the downstream ICD effect (**Figure 5**). Taken together, the discovery of immunogenic lncRNAs supports the elucidation of the complex ICD mechanism and guides ICD-related lncRNAs to illuminate cancer immunotherapy.

#### 3.2.3 CircRNAs and ICD

The evolutionary conservation of circRNAs generally suggests that they perform certain essential functions. Current studies have uncovered that circRNAs serve a regulatory function in physiological and pathological processes through various pathways, mainly including acting as miRNA sponges, interacting with proteins, coupled with regulating transcription, splicing and translation (89). In the tumor microenvironment, circRNAs can intervene in the activity of immune-related cells or the expression of programmed death-ligand 1(PD-L1) to mediate tumor immune surveillance (90). For example, cancer cell-derived exosomal circUHRF1 exerted tumor immunosuppression by inducing NK cell exhaustion and reducing sensitivity to programmed cell death protein 1 (PD-1) blockade immunotherapy in hepatocellular carcinoma (HCC) (91); increased circFAT1 coordinately controlled cancer stemness and immune evasion through promoting signal transducer and activator of transcription 3 (STAT3) activation and inhibiting CD8^+^ T cell infiltration into the tumor microenvironment (92). Thus, the evidence above suggests that targeting immune-related cells and the PD-1/PD-L1 pathway through circRNAs could be a prospective orientation for cancer immunotherapy.

Furthermore, immunogenic circRNAs can act as tumor antigens or be mediated to express tumor-specific antigens for ICD-related immune responses. CircFam53b is a tumor-specific circular RNA that encodes a highly immunogenic cryptic antigen with a strong affinity for human leukocyte antigen (HLA), which causes massive infiltration of CD8^+^ T cells and induces ICD-related immune response (93). Vaccines based on circFam53b or its encoded cryptic antigen can promote antigen presentation and motivate ICD-related immunity, thereby effectively controlling the progression of breast cancer and melanoma. Accordingly, vaccines based on tumor-specific circRNAs possess potent ICD effects, and further studies are required to evaluate the potential of immunogenic circRNAs in enhancing clinical outcomes.

## 4 Targeting strategy and multipronged approaches

### 4.1 Strategies for suppressing ICD-related ncRNAs

In this section, we review the pros and cons of the therapeutic loss-of-function strategies based on ICD-related ncRNAs. In particular, we focus on antisense oligonucleotides, which have emerged as a leading candidate in ncRNA therapeutics due to their favorable pharmacological properties and programmable design. We also describe two competing drug modalities: small interfering RNAs (siRNAs) and miRNAs.

#### 4.1.1 Antisense oligonucleotides

Antisense oligonucleotides represent the initial technology in using oligonucleotides for disease treatment and have been the most successful approach to gene therapy (94). They complementarily pair with messenger RNA through single-stranded nucleotides, blocking specific gene expression and protein synthesis (95). By designing different antisense oligonucleotides, it is possible to inhibit the functions of various messenger RNAs, thereby achieving the goal of disease treatment. The first antisense oligonucleotide drug approved is Fomivirsen, which was approved by the food and drug administration (FDA) in 1998 for the treatment of cytomegalovirus retinitis in acquired immune deficiency syndrome (AIDS) patients (96). In cancer immunotherapy, antisense oligonucleotides have the potential to directly target ncRNAs by designing precise molecules that reduce or inhibit the expression of specific ICD-related ncRNAs, thereby impacting immunogenicity.

#### 4.1.2 siRNA-based therapy

siRNAs are double-stranded RNA molecules with 20-25 nucleotides in length, containing a guide strand and a passenger strand (97). The functional mechanism of siRNA drugs mainly involves the RNA interference (RNAi) pathway, which identifies and degrades complementary RNA, thereby silencing the expression of related proteins (98). At present, significant advances in the formulation of siRNAs have enabled long-term effectiveness *in vivo*, allowing dose adjustments every 6 months (99). In this context, siRNA offers the potential to target ICD-related ncRNAs directly, thereby impacting cancer immunotherapy. This involves designing precise molecules to decrease the expression of ncRNAs associated with ICD negatively and enhance the ICD effect.

#### 4.1.3 miRNA-based therapy

Therapeutic strategies based on miRNA are mainly divided into two categories: miRNA antagonists and miRNA mimics (74). MiRNA antagonists are chemically synthesized miRNA passenger strands, also known as anti-miR or antagomiR (76). MiRNA antagonists can complement and pair with the active strand of miRNA with high affinity to form new double-stranded miRNA, which inhibits the function of miRNA through the RISC degradation pathway (76). The first miRNA antagonist to undergo clinical research is Miravirsen, an inhibitor that complements the 5’ end of miR-122 (100). Phase I and Phase II clinical trials have demonstrated the efficacy of Miravirsen in treating hepatitis C without serious side effects (101). According to this, we can design miRNA antagonists that target ICD-related ncRNAs to expand cancer immunotherapy by amplifying the immunogenicity of tumor cells.

In general, siRNA and antisense oligonucleotides have different characteristics in ncRNAs-mediated therapy, and it is difficult to foresee which one will become the dominant technology. The selection of each strategy should consider the unique biological characteristics and mode of action of the ICD-mediated ncRNAs comprehensively. Antisense oligonucleotides and siRNAs have the potential to directly target ncRNAs to reduce or inhibit the expression of specific ICD-related ncRNAs, thereby impacting the ICD effect. On the other hand, miRNA antagonists offer an alternative therapeutic approach by disrupting the interaction between ICD-related ncRNAs and their molecular partners or directly intervening in the structure of ICD-related ncRNAs.

### 4.2 ICD-related ncRNAs delivery system

Although some therapeutic ncRNAs have been approved for disease treatment, the clinical application of ncRNAs still faces the following challenges: (1) the naked single-stranded RNA molecules are easily degraded by nucleases in the physiological environment; (2) the low delivery efficiency and off-target effects are limitations of ICD-related ncRNAs; (3) ncRNAs are biological macromolecules with negatively charged, making it difficult to penetrate the anionic lipid bilayer and enter cells. In addition, it is worthy of attention to explore how to rationally harness the immunogenicity of ncRNAs in clinical applications to expand the efficacy of ICD. Therefore, it is necessary to design appropriate strategies for delivering ICD-related ncRNAs to the tumor microenvironment to fully realize the potential for cancer immunotherapy.

#### 4.2.1 Exosome-mediated ncRNAs delivery

Exosomes are nanoscale vesicles secreted by cells to facilitate intercellular communication through delivering specific “cargo”, such as proteins, lipids, and nucleic acids (102). Properties of exosomes, such as excellent biocompatibility, minimal cytotoxicity, and high stability in circulation, make them attractive as ideal delivery systems for cancer therapy (103). For example, engineered exosomes can effectively encapsulate miR-140 and deliver it to chondrocytes, which has been shown to alleviate the progression of osteoarthritis (104). MiR-140 functions as an immune mediator that contributes to the suppressive effect of tumors and serves as a potential molecular target in cancer immunotherapy (105). Mechanistically, the immunomodulatory effects of miR-140 are associated with increased infiltration of cytotoxic T cells and decreased infiltration of myeloid-derived suppressor cells, suggesting that miR-140 is an ICD-related ncRNA (106). Accordingly, delivering ICD-related ncRNAs via engineered exosomes is theoretically feasible and is expected to inhibit tumor development through enhancing the immunogenicity of tumor cells. In addition, engineered exosomes have been shown to specifically induce ICD in drug-resistant tumors, thereby bypassing the apoptotic pathway to eliminate tumor-resistant cells efficiently (107). Therefore, ICD-related ncRNAs combined with exosome delivery vesicles have the potential to overcome the challenges and dilemmas of cancer immunotherapy.

#### 4.2.2 Lipid delivery systems for ICD-related ncRNAs

Lipid-based ncRNAs delivery systems include anionic liposomes, neutral liposomes, cationic liposomes, and lipid particles (108). Encapsulating ncRNAs in anionic or neutral liposomes can prevent ncRNAs from nucleases-mediated degradation while remarkably improving their biocompatibility and pharmacokinetic properties (109). Although cationic liposomes have lower biocompatibility compared to other liposomes, they can enhance the enrichment of ncRNAs in the tumor microenvironment and facilitate the uptake of tumor cells (108). The current focus of liposome-delivered ncRNAs is primarily on miRNAs. For example, miRNA-29b, miRNA-101, miRNA-122, miR-181a, and miR-2000 are ncRNAs that have been successfully encapsulated in liposomes and have been shown to exert anti-tumor activity in lung cancer, hepatocellular carcinoma, and blood malignancies (110). Moreover, lipid particles-encapsulated siRNA targeting the cell-cycle proteins polo-like kinase 1 (PLK1) have been used in phase I and II clinical trials for patients with advanced solid tumors (111).

When it comes to lipid delivery systems, researchers are focusing on their adaptability and modifiability. Researchers anticipate that the engineered liposomes will not be selective for the encapsulated ncRNAs and will transport different ncRNAs with equal efficiency. In this context, ICD-related ncRNAs are expected to be efficiently delivered by liposomes to optimize cancer immunotherapy.

#### 4.2.3 Other delivery systems for ICD-related ncRNAs

Polymer nanocarriers are biodegradable colloidal delivery systems widely used for the delivery of drug and ncRNAs (112). For example, CALLA-01, a water-soluble polycationic nanocarrier containing siRNA based on cyclodextrin polymer, is the first polymer nanoparticle used in clinical trials for tumor-targeted siRNA delivery (113). Engineered polymer nanoparticles function as immune adjuvants to enhance anti-tumor immune responses and have been demonstrated in tumor models (114). Using polymer nanoparticles to deliver ICD-related ncRNAs may achieve high encapsulation efficiency, sustained release behavior, and broaden the therapeutic effect of ICD.

The nucleic acid-conjugate delivery systems directly couple ncRNAs to the delivery material for obtaining single-component drug delivery systems with unequivocal properties (115). Given its inherent immunogenicity, tumor antigens offer an optional carrier for co-delivering ICD-related ncRNAs to create customized agonists that target anti-tumor immune responses.

In addition, although chemical modification cannot directly serve as a delivery system for ncRNAs, it can obviously improve the properties of the ncRNAs (116). Reasonable chemical modification can enhance the physiological stability and targeting of ncRNAs (117). Importantly, chemical modifications can improve the immunogenicity of ncRNAs, which is beneficial for stimulating anti-tumor immune response. Therefore, modifying ICD-related ncRNAs to amplify the immunogenic effect is a novel avenue to induce ICD and enhance cancer immunotherapy.

### 4.3 Combined therapeutic strategy based on ICD-related ncRNAs

Utilizing gene therapy based on ICD-related ncRNAs can enhance cancer immunotherapy by strengthening immunogenic effects. However, due to the limited immunogenicity of monotherapy, it is necessary to combine it with other methods to induce ICD and further enhance the effectiveness of cancer immunotherapy. To date, studies have shown that various physical stimuli (such as photodynamic therapy, ionizing radiation, near-infrared photoimmunotherapy, high pressure, and hyperthermia) can induce the immunogenic death of tumor cells, which represents a new direction in exploring cancer immunotherapy for the future (24). Therefore, ICD-related ncRNAs combined with other pathways that induce immunogenic death of tumor cells could boost immunogenicity to expand anti-tumor immune responses, thereby improving the immunotherapeutic effect of tumor patients.

Conventional chemotherapy drugs are tremendously cytotoxic and concentrate on eliminating dividing tumor cells (118). The therapeutic strategy of inducing cell death appears reasonable, as inhibiting tumor development through programmed cell death (e.g., apoptosis, necroptosis, autophagy, ferroptosis, pyroptosis, etc.) has been extensively researched (119). However, the therapeutic strategy for modulating ICD in cancer immunotherapy differs from conventional strategies of triggering tumor cell death. The therapeutic strategy targeting ICD-related ncRNAs regulates multiple DMAPs in the tumor microenvironment to indirectly eliminate tumor cells, indicating that it could be utilized as a combined approach for cascade-enhanced tumor suppression. Hence, multi-modal therapeutic strategies based on ICD and other programmed cell death could eliminate tumor cells in a multipronged manner and effectively prevent tumor progression.

## 5 Challenges and perspectives

Emerging research in cancer immunotherapy attempts to reveal how ncRNAs and ICD synergistically enhance tumor immunogenicity and induce antitumor immune responses. However, much remains to be discovered about unknown ncRNAs, particularly ICD-related ncRNAs and their functional mechanisms. Even for the widely known ncRNAs, their roles in ICD and executed mechanisms could be altered with spatial-temporal adjustment. Therefore, efforts still need to analyze the expression levels and spatiotemporal regulation of ICD-related ncRNAs. With the continuous progress of sequencing technology, we expect that single-cell RNA sequencing will be integrated with new high-throughput techniques, such as spatial transcriptomics, to identify specific ICD-related ncRNAs with high sensitivity and precision. Moreover, existing research suggests that ICD plays a multifaceted role in the immune system, partially elucidating the ambiguity of ICD in cancer immunotherapy (120). This again underscores the requirement for further studies on the context-dependent role of ICD-related ncRNAs in cancer immunotherapy. In addition, evidence straightly supporting the effect of the ICD-related ncRNAs on cancer immunotherapy is currently unavailable but is theoretically reasonable. Accordingly, *in vivo* experiments and prospective clinical research are urgently needed to evaluate the clinical significance of ICD-related ncRNAs in cancer immunotherapy.

Immunogenic death is one of the modes of tumor cell death that can also occur throughout the body (121). Therefore, the systemic effects on the host immune system should be cautiously considered when conducting cancer immunotherapy based on ICD-related ncRNAs. Inspired by drug delivery systems, engineered ncRNAs with specific and efficient targeting capabilities are more optimized therapeutic tools. Furthermore, ICD is different from apoptosis induced by conventional chemotherapeutic drugs, indicating that ICD-related ncRNAs could be utilized as a combination strategy to improve the effectiveness of tumor eradication. Hence, precise identification of patients most likely to benefit from ICD-related ncRNAs-mediated cancer immunotherapy and enhanced tumor eradication through single or multimodal therapeutic strategies represent opportunities for antitumor therapy.

While ncRNAs have been shown to enhance ICD and improve the effectiveness of cancer immunotherapies, recent research suggests that some ncRNAs may confer resistance to ICD induction and chemotherapy (122, 123). Here, we provide a summary of how ncRNAs could contribute to such resistance: (1) suppressing the expression and/or release of DAMPs, thereby reducing ICD and contributing to resistance against cancer therapies; (2) inhibiting the expression of drug efflux pumps (e.g., ATP-binding cassette transporters) and anti-apoptotic proteins (e.g., Bcl-2), thereby reducing the susceptibility of tumor cells to chemotherapy and ICD; (3) constructing an immunosuppressive tumor microenvironment, thereby reducing the efficacy of ICD and chemotherapy.

Taken together, the future development of ICD-related ncRNAs will focus on the following aspects: (1) decoding more information about ncRNAs and identifying key ncRNAs involved in the ICD process; (2) establishing standardized ICD evaluation criteria to detect the effects of ICD-related ncRNAs; (3) analyzing the crosstalk between ncRNAs and ICD and devising personalized therapeutic strategies based on ICD-related ncRNAs; (4) developing the design, synthesis, modification and delivery technologies of ncRNAs to reduce off-target effects and improve the performance of ICD-related ncRNAs; (5) combining with other therapeutic strategies to improve the antitumor immune response and the effectiveness of tumor eradication; (6) classifying tumor patients according to genetic mapping and assessing the suitability of ICD-related ncRNAs for individual patients; (7) constructing an algorithm and/or ncRNA network to predict prognostic and therapeutic responses.

## 6 Conclusion

In summary, ICD is now recognized as a complex cell death program that serves as a cornerstone of cancer immunotherapy. ICD-related ncRNAs present therapeutic potential as immunoregulation signals, providing opportunities to enhance antitumor immune responses in difficult-to-treat malignancies. Furthermore, in-depth research of ICD-related ncRNAs is advantageous in monitoring and evaluating the antitumor immune response. In the future, targeting ICD-related ncRNAs through single or combined pathways should be considered as a new paradigm for cancer immunotherapy and a complement to precision medicine, which may improve the outcomes of current cancer treatments.
